# A longitudinal study to examine the influence of farming practices and environmental factors on pathogen prevalence using structural equation modeling

**DOI:** 10.3389/fmicb.2023.1141043

**Published:** 2023-04-06

**Authors:** Martine Ferguson, Chiun-Kang Hsu, Christopher Grim, Michael Kauffman, Karen Jarvis, James B. Pettengill, Uma S. Babu, Lisa M. Harrison, Baoguang Li, Alice Hayford, Kannan V. Balan, Josefina P. Freeman, Gireesh Rajashekara, Erin K. Lipp, Ralph Scott Rozier, Anne Marie Zimeri, Laurel S. Burall

**Affiliations:** ^1^Office of Analytics and Outreach, Center for Food Safety and Applied Nutrition, U.S. Food and Drug Administration, College Park, MD, United States; ^2^Office of Applied Safety and Research Assessment, Center for Food Safety and Applied Nutrition, U.S. Food and Drug Administration, Laurel, MD, United States; ^3^Center for Food Animal Health, The Ohio State University, Wooster, OH, United States; ^4^Department of Environmental Health Science, University of Georgia, Athens, GA, United States

**Keywords:** farm management practices, fresh produce, *Listeria*, *Salmonella*, *Campylobacter*, STEC, *Arcobacter*, structural equation modeling

## Abstract

The contamination of fresh produce with foodborne pathogens has been an on-going concern with outbreaks linked to these commodities. Evaluation of farm practices, such as use of manure, irrigation water source, and other factors that could influence pathogen prevalence in the farming environment could lead to improved mitigation strategies to reduce the potential for contamination events. Soil, water, manure, and compost were sampled from farms in Ohio and Georgia to identify the prevalence of *Salmonella*, *Listeria monocytogenes* (*Lm*), *Campylobacter*, and Shiga-toxin-producing *Escherichia coli* (STEC), as well as *Arcobacter*, an emerging human pathogen. This study investigated agricultural practices to determine which influenced pathogen prevalence, i.e., the percent positive samples. These efforts identified a low prevalence of *Salmonella*, STEC, and *Campylobacter* in soil and water (< 10%), preventing statistical modeling of these pathogens. However, *Lm* and *Arcobacter* were found in soil (13 and 7%, respectively), manure (49 and 32%, respectively), and water samples (18 and 39%, respectively) at a comparatively higher prevalence, suggesting different dynamics are involved in their survival in the farm environment. *Lm* and *Arcobacter* prevalence data, soil chemical characteristics, as well as farm practices and weather, were analyzed using structural equation modeling to identify which factors play a role, directly or indirectly, on the prevalence of these pathogens. These analyses identified an association between pathogen prevalence and weather, as well as biological soil amendments of animal origin. Increasing air temperature increased *Arcobacter* and decreased *Lm*. *Lm* prevalence was found to be inversely correlated with the use of surface water for irrigation, despite a high *Lm* prevalence in surface water suggesting other factors may play a role. Furthermore, *Lm* prevalence increased when the microbiome’s Simpson’s Diversity Index decreased, which occurred as soil fertility increased, leading to an indirect positive effect for soil fertility on *Lm* prevalence. These results suggest that pathogen, environment, and farm management practices, in addition to produce commodities, all need to be considered when developing mitigation strategies. The prevalence of *Arcobacter* and *Lm* versus the other pathogens suggests that multiple mitigation strategies may need to be employed to control these pathogens.

## 1. Introduction

Foodborne illness associated with the consumption of fresh produce, particularly leafy greens, tomatoes, cantaloupes, tree fruit, and peppers, continues to be a major health concern with numerous outbreaks linked to these commodities (Iwu and Okoh, 2019). This diversity in product type suggests that common risk factors should be considered when evaluating farming practices or developing on-farm risk-mitigation approaches, not just produce-specific factors. Numerous studies have identified bacterial pathogens in agricultural environments, clearly indicating the potential for contamination of produce during cultivation and harvest (Cooley et al., 2007; Jeyaletchumi et al., 2011; Micallef et al., 2012; Strawn et al., 2013a,b; Marine et al., 2015; Belias et al., 2021). An evaluation of bacterial pathogens linked to foodborne illness, conducted by the Interagency Food Safety Analytics Collaboration (IFSAC) implicated four key bacterial pathogens, non-typhoidal *Salmonella*, *E. coli* O157, *Listeria monocytogenes (Lm)*, and *Campylobacter* spp., based on frequency and severity of illness. These findings agree with other efforts identifying these four bacterial pathogens as the primary concerns for foodborne illness (Scallan et al., 2011; IFSAC, 2018).

Non-typhoidal *Salmonella* and Shiga-toxin-producing *E. coli* (STEC) are associated with a large proportion of the burden associated with foodborne outbreaks. *Salmonella* causes an estimated one million cases and is responsible for over a third of the hospitalizations, annually (Scallan et al., 2011). Furthermore, from data derived from an active and passive surveillance study between 2000 and 2008, it was estimated that STEC cause roughly 265,000 illnesses annually in the United States (US), resulting in over 3,600 hospitalizations and 30 deaths (Scallan et al., 2011). Many serovars of non-typhoidal *Salmonella* and STEC are also enteric pathogens to a wide range of wild and domesticated animals (Hoelzer et al., 2011; Persad and LeJeune, 2015). Due to this burden, research has evaluated the potential of these pathogens to survive in manure and in the field environment (Himathongkham et al., 1999; Sharma and Reynnells, 2016; Hruby et al., 2018; Gu et al., 2019; Sharma et al., 2019). The presence of animal hosts in the pre-harvest environment and the application of manure or compost as a soil amendment may facilitate the dissemination and survival of *Salmonella*, as well as other pathogens, in soil (Shah et al., 2019; Bardsley et al., 2021).

*Lm* is well-characterized as a saprophytic bacterium and considered ubiquitous in soil and the environment and the causative agent of invasive listeriosis, a comparatively rare illness that causes ∼19% of foodborne illness deaths (Ivanek et al., 2006; Scallan et al., 2011; Vivant et al., 2013; Iwu and Okoh, 2019). Due to its established role as a saprophytic organism, *Lm* may be better adapted to soil and soil microbiome competition than STEC and *Salmonella* (Freitag, 2009). Data have shown an association of *Lm* with wet soil environments and rain, but longitudinal studies evaluating *Lm* in agricultural fields are limited (Locatelli et al., 2013; Falardeau et al., 2018; Harrand et al., 2020). Watershed studies have also demonstrated that *Lm*, if found in water and sediment samples, could be introduced into the field environment if contaminated water is used for irrigation (Gorski et al., 2014; Stea et al., 2015). Additionally, it has been demonstrated that soil type can impact *Lm* survival and/or growth (Dowe et al., 1997; Jiang et al., 2004; Brennan et al., 2014). Furthermore, manure usage can further support *Lm* survival, depending on manure type (Dowe et al., 1997; Jiang et al., 2004; Brennan et al., 2014). These observations indicate the need for risk assessments using a variety of conditions to better understand the factors at play in *Lm* agricultural prevalence.

*Campylobacter* species are the leading cause of human bacterial gastroenteritis (EFSA and ECDC, 2021), and are predominantly associated with the consumption and handling of improperly cooked poultry, raw milk, and water. However, *Campylobacter* outbreaks have been associated with row crops and other produce, representing 6 and 1.7% of *Campylobacter* outbreaks, respectively (Gardner et al., 2011; IFSAC, 2018; Sher et al., 2021). Similarly, *Arcobacter* spp., which belong to the family *Campylobacteraceae* are classified as a serious hazard to human health by the International Commission on Microbiological Specifications for Foods (ICMSF) (Hoa et al., 2006; Ramees et al., 2017). Recently, *Arcobacter* prevalence was reported in fresh produce (Abay et al., 2022, Ma et al., 2022), and they are widely distributed among animals and environmental water (Collado and Figueras, 2011; Ramees et al., 2017; Niedermeyer et al., 2020). Furthermore, *Arcobacter* spp. have been associated with foodborne outbreaks (Lappi et al., 2013; EFSA and ECDC, 2021; Uljanovas et al., 2021). There are few longitudinal studies assessing *Campylobacter* prevalence in farm environments and in produce, and none for *Arcobacter* (Chai et al., 2009; Guevremont et al., 2017). Thus, there is limited understanding of the presence of these pathogens in the produce farm environment, and transmission to humans.

Farm practices, including irrigation, soil amendment types, pest control, and worker hygiene, have been highlighted as potential sources of pathogen introduction to the farm environment and/or produce. For example, biological soil amendments of animal origin (BSAAO) have been associated with higher risk of pathogen introduction, in particular, when not properly treated prior to use (i.e., composted), (Iwu and Okoh, 2019). In addition to the application of BSAAO to the field, dispersal of pathogen-containing fecal material in the produce preharvest environment could occur *via* surface water runoff and/or animal intrusion (Atwill et al., 2012; Pandey et al., 2014; Sharma and Reynnells, 2016; Alegbeleye and Sant’Ana, 2020). Enteric pathogens have also been detected in source water or distribution systems of irrigation water used in produce farms in multiple US coastal states (Gu et al., 2013, 2020; Draper et al., 2016; Allard et al., 2019; Weller et al., 2020).

This study evaluated pathogen prevalence and survival in the pre-harvest environment in two US geographic regions. These data provide critical information to compare and evaluate the role of soil characteristics, climate, and farm management practices on pathogen prevalence, and improve the understanding of pathogen ecology in agricultural settings. This knowledge, especially when coupled with commodity-specific risk assessments, may aid in the development of targeted mitigation strategies to enhance the microbial safety of fresh produce.

## 2. Materials and methods

### 2.1. Farm characteristics

The study was initiated in the 2018 growing season as a small pilot study to evaluate potential regional differences in pathogen prevalence, alongside evaluating the effect of some farm management practices on pathogen prevalence. In 2019, the study was expanded to include more pathogens, increased sampling, and the contribution of irrigation water sources. The study ended in 2020 due to COVID-19 restrictions preventing monthly sampling, with the heaviest restrictions coinciding with amendment application.

Farms were recruited in both Georgia (GA) and Ohio (OH) by voluntary agreement of the farmers. All farms were blinded for the purposes of this study. Four farms in GA elected to participate in 2018, two of which also participated in 2019. Two new farms participated in GA in 2019, for a total of six participating farms in GA over both years. In each year a single, but different, farm was removed from the study due to circumstances unrelated to the study. All GA farms were certified organic farms, growing mixed commodities. During the 2018 growing season, limited sampling occurred on three GA farms/fields, with samples (amendment and soil) collected pre-amendment, post-amendment, mid-season and at harvest. In 2019, to expand the study as noted earlier, fields associated with certified organic farms were selected based on amendment type (BSAAO or green compost). Green compost was a BSA of non-animal origin, e.g., plant-based. BSAAO samples included both raw and composted manure samples, which were not analyzed separately in this study due to the limited number of composted manure samples. Composite samples were collected monthly from each field, starting prior to amendment application, and continuing through harvest. GA fields were subdivided into four sections that were sampled at each site visit. Some of these sections had differing amendment types but the sections were defined by the commodity grown within each section. Each of these sections were considered separate fields in the analysis.

In OH, 18 farms, using a variety of farm management approaches, were similarly recruited with six farms for each amendment type, dairy manure (DM)-amended, poultry manure (PM)-amended, and non-BSAAO-amended. The farm management approaches in OH ranged from conventional to organic with most using a hybrid approach. None of the OH farms were certified organic. The study included fields in rotation (i.e., fields that were cultivating a crop with the intention of restoring soil health). As these fields had crops growing and were not fallow, these fields are referred to as rotating fields. The OH farms are characterized as small, ranging from 80 to 120 acres; each grew mixed commodities on a staggered schedule, and often had animals residing on the farm, including work animals, or on neighboring farms.

### 2.2. Metadata collection

Metadata on farm management practices were collected *via* an interview with each farmer at the end of the growing season. Observations were recorded where available but complete metadata were not always available due to differences in reporting by farmers. These metadata include the use of organic management practices, use and type of fertilizers and pesticides, presence of domestic animals, evidence of intrusion from wild animals, and other events of note.

Daily meteorological data, provided by the Ohio Agricultural Research and Development Center weather system, were obtained from a local weather station in OH, located within an ∼24 km radius of all sampled fields. The data included total liquid precipitation (inches of accumulation, collected midnight to midnight, melted in case of ice or snow), average/minimum/maximum air temperature (^o^F, average determined with the 24 hourly readings for each day), global solar radiation measured at 6 m (Langleys units, 1 Ly = 1 cal/cm^2^, sum of 5 min readings in 24 h, average/minimum/maximum soil temperature (^o^F, top 2 and top 4 inches) (Supplementary Table 1). Wind speed and relative humidity were excluded from this study due to errors in data collection.

In GA, daily meteorological data were collected from the University of Georgia Climatology Research Laboratory and included maximum rain gauge (inches), i.e., total precipitation, average air temperature (^o^F), average solar radiation (watts per square meter), and average 10 min wind gust (mph), i.e., wind speed (WS) (Supplementary Table 2). Soil temperature was not collected in GA. Solar radiation was measured differently in GA and OH. To correct for this, when comparing GA and OH weather, solar radiation data were standardized by subtracting the observed value from the observed state mean value and then dividing by the observed state standard deviation.

### 2.3. Sample collection and processing

#### 2.3.1. Biological soil amendments and manure

A single composite biological soil amendment (BSA, green compost) or BSAAO sample, consisting of 3–4 sub-samples, was taken from the compost or manure at the time of application to the field. DM, which was applied only to fields in OH, was comprised of a combination of straw/corn fodder and dairy heifer manure that had accumulated for several months, in the pen housing the animals, before being transferred to a manure spreader. Three to four sub-samples were collected from one of the manure spreaders and combined into a single composite sample for testing. PM was purchased by the farmers from a local broiler house and delivered to the farm as a pile with no covering. Time between delivery and sample collection varied from farm to farm. In the OH region, monthly DM samples were collected from farms using DM amendments to evaluate the longitudinal pathogen prevalence in the herds beyond the manure that was applied to the field. These samples were formed by combining 8–10 fresh manure pats into a composite DM sample and were often found in the housing facilities or grass pasture areas. Samples were shipped at temperatures consistent with environmental conditions to minimize changes. The received samples were aliquoted (25 g) aseptically into sterile, filter Whirl-Pak^®^ bags and then processed for pathogen detection. The samples were tested for the presence of five pathogens, *Lm*, *Arcobacter*, *Salmonella*, STEC, and *Campylobacter*. STEC analyses were added in the 2018–2019 growing season as part of the expansion mentioned in Section “2.1 Farm characteristics.”

#### 2.3.2. Water

In OH and GA, water samples were collected in 2019 from the water source (pond, well, or creek/stream) and from the end of the drip tape. Stream water samples were collected within 3 to 4 ft from the edge of the stream and near the irrigation system’s pump. Pond water samples were collected 3 to 10 ft from the shore and near the irrigation pump. Well source water samples were either sampled at the well head, directly from the pump, or after the water had travelled through an irrigation pipe from the well to the field. While all GA fields used well water for irrigation, water samples were also collected from surface water adjacent to the GA farms.

Ten liters of source water were filtered using Modified Moore Swabs (MMS) for pathogen testing including *Lm*, *Arcobacter*, *Campylobacter*, *Salmonella* and STEC, as well as for 16S rRNA gene sequencing analysis. Additionally, two liters of water samples, collected from the end of the dripline, were filtered through MMS. The MMS were maintained at refrigeration prior to pathogen testing. All MMS were then bisected using a disposable sterile scalpel to ensure open exchange during processing. The bisected MMS were transferred to sterile, filter Whirl-Pak^®^ bags and 250 mL of sterile distilled water was added to each. The MMS were manually massaged for 15–30 s to evenly distribute the swabs within the Whirl-Pak^®^ bags and then stomached at 300 rpm for 2 min in 30 s increments, with manual massaging between intervals to redistribute the MMS. Aliquots of 25 mL were collected from each sample, with manual agitation between samples to redistribute any sediment that had settled and processed according to the appropriate pathogen protocol.

#### 2.3.3. Soil

In the 2017–2018 season, four composite samples were collected from each field representing pre-amendment, post amendment, mid-season, and harvest time. Preliminary analysis indicated more frequent sampling was needed. For this reason, in the 2018–2019 growing season, one composite sample was collected monthly per field in OH and GA. Each composite sample was comprised of three random sub-samplings from different locations in the field, all collected from between the rows of growing crop, which were combined as a composite sample weighing ∼2 kg. Each sub-sample entailed collection of soil from an area of 6” x 6” x 1” (width x length x depth) by spade, treated with 70% ethanol between sampling, with visible debris removed.

The soil samples were shipped and processed for pathogen detection and 16S rRNA gene sequencing analysis in the same way described above for the BSA and manure samples. A portion (∼0.5 kg) of soil samples, collected pre-amendment, post-amendment, mid-season, and at harvest, was shipped to The Maine Agricultural and Forest Experiment Station Analytical Laboratory and Maine Soil Testing Service^1^ for analysis by their comprehensive soil test using the Mehlich 3 extraction method (Rutter et al., 2022). This comprehensive soil test assessed pH, total organic matter, available nitrogen (nitrate plus ammonium), phosphorus, potassium, calcium, magnesium, sulfur, boron, copper, iron, manganese, sodium, and zinc. Separately, 25 g samples were processed for pathogen detection and an additional 250 mg was used for DNA extraction for 16S rRNA sequencing gene analysis.

### 2.4. Pathogen detection methods

#### 2.4.1. *Listeria monocytogenes*

Cultural enrichment and detection of *Lm* largely followed ISO 11290, with one modification (Gnanou Besse et al., 2019). Ferric citrate, which serves as a screening tool, was omitted from the medium as early testing found limited association with *Lm* presence (data not shown) (Donnelly, 2002; Leclercq, 2004; Parsons et al., 2019). Antibiotics were obtained from MilliporeSigma (Sigma-Aldrich^®^, St. Louis, MO). Samples were processed according to ISO 11290 with manual massage, due to the presence of rocks. Detection was performed by plating 10 μL on Rapid L’mono (RLM) agar (Bio-Rad, Hercules, CA), according to manufacturer’s procedures, and then subcultured on RLM to mitigate media changes due to background flora. Presumptive *Lm* colonies were then cultured on Brain Heart Infusion (BHI) agar (Becton, Dickinson and Company, Sparks, MD) and confirmed *via* qPCR analysis (Burall et al., 2021).

#### 2.4.2. *Arcobacter* and *Campylobacter*

Soil and manure samples were pre-enriched in 75 mL Bolton broth (ThermoFisher Scientific Oxoid limited, Hampshire, UK) containing amphotericin B (5 mg/L) in filter Whirl-Pak^®^ bags at 37°C for 3–4 h. This was followed by enrichment of 3 mL pre-enriched sample in 7 mL Bolton broth containing 5 mg/L amphotericin B, 20 mg/L vancomycin, 5 mg/L cefsulodin, and 10 mg/L trimethoprim for 48 h under microaerobic (MA) conditions (5% O_2_, 7.6% CO_2_, 7.6% H_2_, 79.2% N_2_). Each MMS rinsate was enriched with 25 mL 2X Bolton broth containing antibiotics (as above), under MA conditions at 37°C for 48 h.

After enrichment, samples were screened microscopically for the presence of spiral to rod or curved shaped bacteria and by PCR using *Campylobacter* (16S rRNA)- and *Arcobacter* (23S rRNA)-specific primers (Linton et al., 1996; Kim et al., 2019). Dilutions of the presumptive positive samples were inoculated (100 μL) onto 0.65 μm mixed cellulose ester filters (Hiett, 2017) placed on Brucella Blood Agar (ThermoFisher Scientific Remel, Lenexa, KS) supplemented with lysed Horse blood (Lampire biological laboratories, Pipersville, PA) for 15 min and/or streaked (20 μL) onto modified charcoal-cefoperazone-deoxycholate (mCCDA) plates. Isolated colonies were screened microscopically and confirmed by PCR.

#### 2.4.3. *Salmonella*

Pre-enrichment of samples was conducted by resuspending each sample in a Whirl-Pak^®^ bag with 225 mL modified buffered peptone water. Each bag was slowly swirled for 1 h in a shaking incubator at 30°C before continuing incubation at 35°C for 24 h (± 2 h). Enrichment for *Salmonella* was performed as described in the FDA Bacteriological Analytical Manual (BAM) (Andrews et al., 2022) with Rappaport-Vassiliadis (RV) medium and Tetrathionate (TT) broth, followed by isolation of black single colonies on XLT-4 agar and confirmation using the VITEK^®^ 2 system (BioMérieux, Inc, Marcy-l’Étoile, France).

#### 2.4.4. Isolation of STEC

Samples were pre-enriched by resuspending each sample in a filter bag with 225 mL modified buffered peptone water with pyruvate (mBPWp) and incubated at 37°C for 5 h (± 1 h). Acriflavin–Cefsulodin-Vancomycin supplement (3 mL) (Feng et al., 2020) was added and the samples were incubated at 42°C for 18 h (± 2 h). Then, two 1 mL replicate aliquots of the enrichments were collected from each sample. One was for screening for STEC by qPCR. The aliquot was centrifuged at 2,500 x g for 3 min for DNA extraction.

The other aliquot was for isolation. When tested positive by PCR, three 10 μL replicates from each culture were streaked onto ChromAgar STEC plates (CHROMagar™, Paris, France) and incubated at 37°C for 18 h. Pink colonies were selected for STEC verification. The colonies were grown in mBPWp in a 96-well plate at 37°C for 5–8 h. Half of the culture was used for DNA purification and the other half was saved as a seed culture for later use. DNA was purified and qPCR analysis was performed to detect STEC as described elsewhere (Li et al., 2017). Wells positive for STEC were tracked to the seed plate, which was used for secondary culture on CHROMagar™ STEC plates for further isolation and purification.

#### 2.4.5. 16S rRNA gene sequencing and analysis

Water samples were centrifuged at 7,200 rpm for 30 min. Cell pellets were then resuspended in DNA/RNA Shield solution (Zymo Research, Irvine, CA) and stored before batch processing for DNA extraction. DNA was extracted from environmental samples with the ZymoBIOMICS DNA Miniprep kit (Zymo Research, Irvine, CA) utilizing the lysis bead bashing tubes and validated lysis protocols on either the FastPrep-24 5G homogenizer (MP Biomedicals, Santa Ana, CA) or a Vortex Genie with Horizontal-(24) Microtube Adaptor. 16S rRNA gene amplicon library preparation and sequencing was performed on the MiSeq benchtop sequencer (Illumina, San Diego, CA) targeting the V4 variable region of the 16S rRNA gene, following the manufacturer’s recommended protocol with the modification of using Omni Klentaq polymerase (DNA Polymerase Technology, St. Louis, MO) in place of KAPA HiFi HotStart Ready Mix, as described elsewhere (Daquigan et al., 2016). The amplification primers used were 515F-Y and 926R (Parada et al., 2016). Nextera XT dual indices were used to allow multiplex sequencing using 600 cycle V3 paired-end chemistry. Each sequencing run contained 60 to 80 multiplexed libraries and estimated equimolar library pools were sequenced at 8 pM. with a 15% phiX spike-in.

Taxonomic classification of 16S V4 amplicon microbiome datasets were processed using an in-house bioinformatics pipeline written in R, Bash, and Python. Preprocessing and classification of reads were performed as follows. First, paired-end reads were merged using FLASH (Magoc and Salzberg, 2011). In cases where reads could not be merged, either due to insufficient overlapping base pairs or poor quality within the overlapping region, read 1 was retained in the dataset. This approach allowed retention of more sequencing data. Next, the paired-merged and read 1 dataset was then processed using USEARCH (Edgar, 2010; Edgar and Flyvbjerg, 2015) to perform quality filtering and prepare reads for taxonomic classification. This included using the fastx_truncate option to remove the 16S rRNA PCR primers, fastq_filter to filter based on a minimum fragment length of 150 bp and ee (expected error) value of 1.0 quality, fastx_uniques to get unique sequences per sample, and unoise3 to check for and remove chimeric sequences. The resulting datasets were then classified with MAPseq (Matias Rodrigues et al., 2017), utilizing their default curated database. Briefly, this was created using NCBI GenBank and RefSeq reference sequence databases, extracting any sequences annotated as ribosomal RNA with 16S or 18S in the annotation. The data presented in the study are deposited in the NCBI repository, accession number PRJNA894200.

### 2.5. Statistical methods

#### 2.5.1. Soil amendment and water

Associations between *Lm*, *Arcobacter*, *Campylobacter*, *Salmonella*, and STEC prevalence and type of soil amendment (DM, PM, and green compost) or water source (surface, including streams and ponds, or well) were assessed using Fisher’s exact test, performed in SAS 9.4 (SAS Institute, Cary, NC). Associations between pathogen prevalence and season were also assessed using Fisher’s exact test. Small sample sizes prohibited multivariate modeling analyses.

#### 2.5.2. OH soil

**FIGURE 1:**
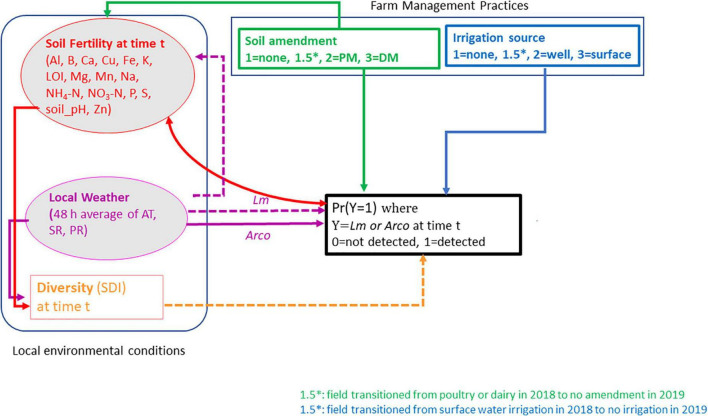
Conceptual model for *Lm* and *Arcobacter* (*Arco*) prevalence in OH soil. Square boxes are observed variables, Farm management practices (soil amendment and irrigation source) and Simpson’s diversity index (SDI). Ovals are latent variables, soil fertility and local weather at time t. Arrows represent the flow of causality. Solid-line arrows indicate positive effects on the *to* variable, dash-line arrows indicate negative effects on the to variable. Double-headed curved arrows are covariances (i.e., no causality). Al, aluminum; B, boron; Ca, calcium; Cu, copper; Fe, iron; K, potassium; Mg, magnesium; Mn, manganese; Na, sodium; P, phosphorus; S, sulfur; Zn, zinc; LOI, loss-on-ignition; PM, poultry manure; DM, dairy manure; AT, air temperature; SR, solar radiation; PR, precipitation.

A structural equation model (SEM) was postulated specifying direct and/or indirect influence of local meteorological data, soil fertility data, farm management practices (soil amendment and source of irrigation water) and taxonomic diversity on the presence of the most prevalent pathogens, *Lm* and *Arcobacter* (Supplementary Tables 1, 2, and 5). The conceptual model, evaluating OH soil data, is outlined in Figure 1, specifying indicators and direction of causality. There were insufficient data to inform the model for *Salmonella*, *Campylobacter*, and STEC. Soil samples that were positive for *Lm* or *Arcobacter* after culture enrichment were considered “positive.” The farm management variables, soil amendment and irrigation water source, were ordinalized with increasing scores corresponding to greater observed *Lm* or *Arcobacter* prevalence. The water data were thus coded: 1 = non-irrigated, 2 = well water-irrigated, 3 = surface water-irrigated. Similarly, the manure data were coded as: 1 = no BSAAO, 2 = PM (including composted PM), and 3 = DM (including composted DM). To account for transition from soil amendment to no BSA amendment in a field from one year to another, i.e., if a field transitioned from PM or DM soil amendment in 2018 to no BSA amendment in 2019, the assigned amendment score was adjusted to 1.5 to account for possible lingering PM or DM effects. Similarly, if a field was irrigated with surface water in 2018 and then transitioned to no irrigation in 2019, that field was assigned an irrigation score of 1.5. This allowed the potential to capture carryover risk from one treatment to another during the SEM analysis.

The soil elements tested in this study were known to influence plant and microbial growth and considered indicators of soil fertility Lines-Kelly, 1992 (Gardner, 1985). These elements, in the collective, are referred to as “soil fertility” in this manuscript, despite no direct assessment of fertility *via* crop growth or yield. Soil fertility was modeled as a latent variable. Soil fertility was defined as the underlying driver of the soil chemical profile values: soil pH, phosphorus, potassium, calcium, magnesium, aluminum, manganese, boron, copper, iron, sodium, ammonium nitrogen (NH_4_-N), nitrate nitrogen (NO_3_-N), sulfur, zinc, and loss-on-ignition, which estimates the organic matter content of the soil. Increasing values of nutrients and soil pH were considered as indicators of increased soil fertility (i.e., positive loadings). In a secondary analysis, both nitrogen analytes (NH_4_-N and NO_3_-N) were analyzed separately from soil fertility to examine their relationship with *Nitrospira*, *Nitrosospira*, *Lm*, and *Arcobacter*. The relative abundance of *Nitrospira* and *Nitrosospira*, the measured values of NH_4_-N and NO_3_-N, and the prevalence of both *Lm* and *Arcobacter* were plotted versus time to assess if prevalence tracked synchronically or asynchronically with the nitrogen analytes and nitrifying bacteria.

Weather was incorporated in the model as a latent variable observed through precipitation (PR), air temperature (AT), and solar radiation (SR), with increasing temperature and radiation values (i.e., positive loading) and decreasing precipitation (i.e., negative loading) representing increasing “weather” across time. Minima and maxima measures were not included to avoid collinearity issues with average daily measures and to reflect the center rather than the extremes of the data. Due to the high collinearity (corr_Pearson_ = 0.93) between soil and air temperatures, soil temperature was not included.

The literature indicated a delayed, or lagged, effect of weather on *Lm* prevalence, with a peak increase in *Lm* prevalence 24 h after a rain event but rain amounts over the preceding 2 days also increased *Lm* prevalence (Weller et al., 2015a,b; Harrand et al., 2020). Based on these data, a 48 h lag was used in this study to evaluate effects linked to precipitation. The term “lag” is used to indicate the timeframe prior to sample collection. The choice of lag (or no lag) or moving average span for the remaining weather parameters was informed by (1) published results and (2) visual examination of loess-smoothed plots.

There are two main components to an SEM. The structural component quantifies potential dependencies (pathways) between the variables in the model. The measurement component quantifies how well the latent variables are represented by the observed indicators (e.g., how well is soil fertility represented by the soil measurements Al, B, Ca, …, Zn). A SEM must have a sound theoretical basis and is only as successful as the researcher-hypothesized *a priori* model, based on researcher knowledge and/or published results.

The hypothesized OH conceptual model, guided by observed manure and water prevalence in Table 1 and prior research, is presented in Figure 1. As the SEM is a model approach driven by theory and prior research, it was postulated that the use of BSAAO, the use of surface water for irrigation, increasing soil fertility, decreasing AT and SR, increasing PR, and decreasing microbiome diversity would result in an increase in *Lm* prevalence. It was also hypothesized that the use of BSAAO, increasing soil fertility, increasing AT and SR, decreasing PR, and decreasing Simpson’s Diversity Index (SDI) would result in an increase in *Arcobacter* prevalence. SDI is a measure of alpha, or within-sample, diversity which takes into account the number of species present and the relative abundance of each species (Roswell et al., 2021). In both approaches, DM was hypothesized to have a larger effect than PM. Microbiome diversity, measured as SDI, was hypothesized to increase with increasing AT, SR, and soil fertility assuming nutrients in the soil can be utilized by bacteria and plants.

**TABLE 1:** Overall pathogen prevalence by sample type and state.

		*Lm*			*Salmonella*			*Campylobacter*			*Arcobacter*			STEC		
		GA	OH	Overall	GA	OH	Overall	GA	OH	Overall	GA	OH	Overall	GA	OH	Overall
Water	Positive n (%)	2 (7)	15 (22)	17 (18)	1 (4)	6 (9)	7 (7)	1 (4)	0	1 (1)	5 (22)	31 (45)	36 (39)	0	2 (3)	2 (2)
	# samples tested	26	69	95	26	69	95	23	69	92	23	69	92	26	69	95
Soil	Positive n (%)	10 (9)	45 (15)	55 (13)	0	0	0	0	0	0	1 (2)	21 (8)	22 (7)	0	1 (0.4)	1 (0.3)
	# samples tested	106	311	417	106	310	416	84	260	344	67	260	327	89	235	324
BSA	Positive n (%)	1 (6)	35 (51)	36 (42)	0	2 (3)	2 (2)	0	21 (31)	21 (24)	1 (20)	23 (34)	24 (33)	0	9 (16)	9 (15)
	# samples tested	18	69	87	18	69	87	18	68	86	5	68	73	5	57	62

BSA refers to all amendment samples, those associated with field application, as well as those not associated with field application. Positive n, the number of samples in which the pathogen indicated was detected; %, percent of samples positive for the pathogen indicated.

Diagonally weighted least squares was implemented using the *sem* function in the R lavaan package, version 0.6.9 (Rosseel, 2012) and the probit link function, Φ () where Φ is the cumulative standard normal distribution function. The model was fit by fixing the variances of the latent variables to unity. As observed weather and soil parameters, SDI, and prevalence data were on different scales, they were standardized to the same scale.

The fit of the SEM was assessed by the Bentler Comparative Fit Index (CFI) and the root mean square error of approximation (RMSEA). Covariances between the soil or weather manifest variables, as suggested by large modification indices (MI), MI > 10 (i.e., $\chi_{d\,f=1}^{2}\,p<0.002$), and fitting the model paradigm, were included in the model. The model was tuned by removing regression parameters and pathways with Wald *z*-test *P*-value > 0.15.

#### 2.5.3. GA soil

**FIGURE 2:**
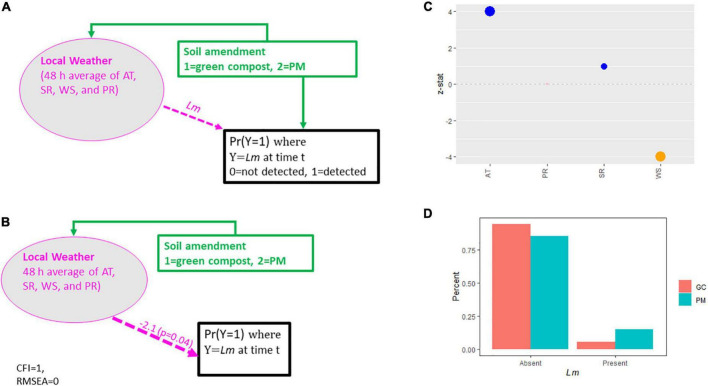
**(A)** Conceptual model for *Lm* prevalence in GA soil. **(B)** Final estimated model for *Lm* prevalence in GA soil. **(C)** Weather effect sizes. GC, green compost. **(D)** *Lm* prevalence in GA soil by soil amendment type. PM, poultry manure; AT, air temperature; SR, solar radiation; PR, precipitation; WS, wind speed; CFI, comparative fit index; RMSEA, root mean square error of approximation.

A more parsimonious SEM was conducted to model *Lm* prevalence in GA soil samples due to insufficient soil fertility panel data. No analysis was performed for the other pathogens, due to limited soil positive samples. Furthermore, all GA fields were well-irrigated, and no GA fields were amended with DM, eliminating irrigation source as a variable and reducing the soil amendment variable to two levels, 1 = green compost, 2 = PM. The hypothesized conceptual model, used to evaluate GA soil data, is presented in Figure 2A with local weather incorporated, as in the OH soil model, with the addition of WS as an indicator. Decreasing AT and SR, increasing PR and WS, and the use of PM amendment were hypothesized to result in increased *Lm* prevalence, similar to the model evaluating OH data.

#### 2.5.4. Bacterial diversity of OH soil

Metataxonomic analysis of the bacterial community was conducted on OH soil samples, targeting the V4 region of the 16S rRNA gene. Families occurring in less than five of the samples were excluded. SDI was calculated, at the family level, using the R *diversity* function in the vegan package Oksanen et al., 2019. SDI is a measure of taxonomic diversity with 1 representing maximum diversity and 0 representing no diversity. SDI was included in the *Lm* and *Arcobacter* prevalence models as the measure of microbiome diversity.

#### 2.5.5. *Lm* and *Arcobacter* co-occurrence in OH soil

Analyses of co-occurrence with *Lm* or *Arcobacter* were conducted to assess which soil genera correlated with the presence or absence of *Lm* or *Arcobacter*. *Lm* culture positive samples that were undetected in the 16S analysis were included by setting a value at half the minimum percent hit versus those positive by 16S analysis. The relative abundances of genera in the *Lm*+ (or *Arcobacter*+) OH soil samples were compared to those in *Lm*- (or *Arcobacter*-) OH soil samples. Non-parametric bootstrap 95% confidence intervals (CI) were calculated for the mean relative abundance of each genus for *Lm*- (or *Arcobacter*-) samples and *Lm*+ (or *Arcobacter*+) samples. CIs were calculated using the *np.boot* function in the R nptest package Helwig, 2021.

## 3. Results

### 3.1. Amendment pathogen prevalence

**FIGURE 3:**
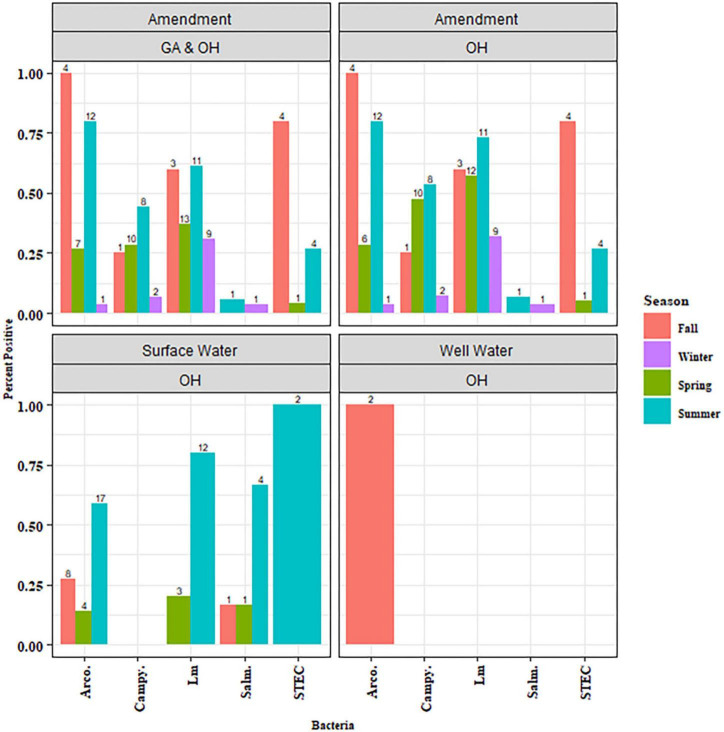
Pathogen prevalence in amendment and water samples by season. Amendment includes the actual BSA applied to fields as well as dairy manure samples that were not associated with field application. Data is plotted to show the percent of the samples tested that were positive for the indicated pathogen evaluated within each season.

Amendment samples (*n* = 87) were tested for the prevalence of *Salmonella*, *Arcobacter*, *Campylobacter*, STEC, and *Lm* (Supplementary Table 3). When comparing pathogen prevalence in OH and GA, all five pathogens were lower in GA amendments (0–20% positive samples) than OH (3–51% positive samples) (*P*-values, not significant), though this may be due to the absence of DM in GA (Table 1). *Lm*, *Salmonella*, and *Arcobacter* were detected in DM and PM samples while *Campylobacter* was only detected in DM samples (Table 2). No pathogens were detected in the green (non-animal) compost samples. The presence of *Campylobacter* and *Lm* was significantly associated with manure type (*P*_*Campylobacter*_ = 0.0002, *P*_*Lm*_ < 0.0001), being most prevalent in DM samples (Table 2). To assess seasonal trends in OH, pathogen prevalence was compared across season (winter, spring, summer and fall). *Lm* (*P* = 0.05) and *Campylobacter* (*P* = 0.001) were most prevalent in the summer and *Arcobacter* (*P* < 0.0001) was most prevalent in the fall, followed by the summer (Figure 3). There were comparatively fewer samples analyzed for STEC, as STEC was added in the second year, and a low prevalence was observed, reducing confidence in the observed results; however, we note that STEC was only detected in DM samples and appeared most prevalent in the fall.

**TABLE 2:** Pathogen prevalence by sample type and subtype.

		Positive n (*%*)						Positive n (*%*)				
		*Lm*	*Salm.*	*Campy.*	*Arco.*	STEC		*Lm*	*Salm.*	*Campy.*	*Arco.*	STEC
Water type (# tested)	All surface* (66)	17 (26)	7 (11)	1 (2)	34 (52)	2 (3)	Drip	7 (18)	1 (3)	0	14 (39)	0
	OH surface only (57)	15 (26)	6 (11)	0	29 (51)	2 (4)						
	All well (29)	0	0	0	2 (8)	0	Source	10 (18)	6 (11)	1 (2)	22 (39)	2 (4)
	OH well only (12)	0	0	0	2 (17)	0						
Soil by amendment or irrigation practice (# tested)	GA no BSA (20)	0	0	0	0	0	Non-irrigated					
	GA poultry-amended (52)	7 (14)	0	0	1 (3)	0	Well-irrigated (108)	10 (9)	0	0	1 (1.5)	0
	GA GC-amended (36)	3 (8.5)	0	0	0	0	Surface-irrigated					
	OH dairy-amended (103)	19 (18)	0	0	10 (11)	1 (1)	Non-irrigated (94)	18 (19)	0	0	7 (10)	1 (1)
	OH poultry-amended (94)	18 (19)	0	0	7 (8)	0	Well-irrigated (125)	14 (11)	0	0	6 (6)	0
	OH no BSA (114)	8 (7)	0	0	4 (4)	0	Surface-irrigated (92)	13 (14)	0	0	8 (10)	0
Amendment (# tested)	Green compost (13)	0	0	0	0	0						
	PM or poultry compost (17)	2 (12)	1 (6)	0	2 (13)	0						
	DM or dairy compost (57)	34 (60)	1 (2)	21 (38)	22 (39)	9 (18)						

All, includes samples from OH and GA; *, stream and pond. *Salm., Salmonella; Campy., Campylobacter; Arco., Arcobacter*; # tested, the number of samples tested; Dairy-amended, soils amended with BSA of dairy origin; Poultry-amended, soils amended with BSA of poultry origin; GC-amended, soils amended with green compost (GC).

### 3.2. Pathogen prevalence in irrigation water

Water samples (*n* = 95) were tested for pathogen prevalence (Table 1, Supplementary Table 4). Thirty-nine samples (*n*_OH_ = 31; *n*_GA_ = 8) were collected from the end of the dripline, and 56 samples (*n*_GA_ = 18, *n*_OH_ = 38) were collected from the water source. *Lm* and *Arcobacter* were by far the most frequently detected pathogens. Comparisons of pathogen prevalence of GA and OH water samples, using Fisher’s exact test, found that *Arcobacter* was more prevalent in water from OH than GA (*P* = 0.05). However, no significant regional differences in prevalence for *Lm*, *Salmonella*, *Campylobacter*, and STEC were observed for water samples (Table 1).

*Arcobacter* was detected in both surface and well source water, with a higher prevalence in surface water (*P*_Overall_ < 0.0001, *P*_OH_ = 0.05), whereas *Lm*, *Salmonella*, *Campylobacter*, and STEC were detected only in surface water (pond or stream) (Table 2). *Campylobacter* and STEC were only detected in source samples whereas *Lm*, *Salmonella* and *Arcobacter* were detected in both source and dripline samples (Table 2).

To assess seasonal trends, pathogen prevalence in OH surface water was compared across seasons (spring, fall, or summer). *Lm* was significantly more prevalent in the summer OH water samples (*P* = 0.02; Figure 3). Some of the subgroups within Figure 3 had sample sizes too small for any conclusions to be drawn and are considered observational. No significant seasonal trend was observed for the other pathogens, either due to the absence of a significant difference or the dataset’s size preventing statistical analysis.

### 3.3. Soil pathogen prevalence

OH soil samples were tested for *Salmonella* (*n* = 310), *Campylobacter* (*n* = 260), STEC (*n* = 235), *Lm* (*n* = 311), and *Arcobacter* (*n* = 260; Table 1). These samples were collected from 21 farms/fields in 2018 and 29 farms/fields in 2019, for a total of 29 different farms/fields across both years with 20 of them sampled in both years. All OH soil samples were negative for *Salmonella* and *Campylobacter*, and only one was positive for STEC. Given the low yield for those three pathogens, prevalence analyses focused on *Lm* and *Arcobacter*. There were 154 (for *Lm*) and 144 (for *Arcobacter*) complete cases, i.e., samples which had complete data on soil fertility, weather, farm management data, and metagenomic to model the pathogen prevalence. Rotating fields (*n*_OH_ = 13) were included in the analysis as non-amended fields (SA = 1) or previously amended fields (SA = 1.5) unless amendment was applied to the rotating field (*n* = 3, DM-amended). Fifteen percent of OH soil samples tested positive for *Lm* and 8% tested positive for *Arcobacter* (Table 1). However, when limited to the 154 (*Lm*) OH soil samples with complete data, 26 (20%) samples tested positive for *Lm*. Likewise, limiting the analysis to the 144 (*Arcobacter*) OH soil samples with complete data, 12 (8%) samples tested positive for *Arcobacter*.

Among the GA soil samples, 106 were tested for *Lm* and *Salmonella*, 89 for STEC, 84 for *Campylobacter*, and 67 for *Arcobacter* (Table 1). While no GA soil samples tested positive for *Salmonella*, *Campylobacter*, or STEC, one (2%) tested positive for *Arcobacter* and ten (9%) tested positive for *Lm*. Of the 10 *Lm*+ GA soil samples, seven were from PM-amended fields (14% of PM-amended fields) and 3 were from green compost-amended fields (6% of green compost-amended fields), indicating increased *Lm* prevalence in GA PM-amended fields versus green compost-amended fields (Table 2). There were 99 complete GA soil cases to model *Lm* prevalence.

*Lm* and *Arcobacter* prevalence of GA and OH soil samples were compared using Fisher’s exact test. *Arcobacter* was more prevalent in OH soil than GA soil (*P* = 0.06), while no differences were observed in *Lm* soil prevalence between the two regions.

Trends of OH soil *Lm* and *Arcobacter* prevalence and GA *Lm* prevalence versus weather were explored, with weather parameters observed at various hours (24, 48, 72, 96, and 120 h) prior to the day of soil collection or averaged across various moving averages (spanning 48, 72, 96, and 120 h) (data not shown). PR averaged over the 48 h prior to and including the day of soil collection correlated most closely with *Lm* prevalence in GA and OH soil and inversely correlated with *Arcobacter* prevalence in OH soil. AT and SR, also averaged over 48 h, inversely correlated with *Lm* prevalence in OH and GA soil and directly correlated with *Arcobacter* prevalence in OH soil. These findings largely agreed with prior literature results for *Lm* (Strawn et al., 2013a,b; Weller et al., 2015a; Harrand et al., 2020). WS averaged over the 48 h prior to and including the day of soil collection was observed to correlate most closely with *Lm* prevalence in GA soil.

#### 3.3.1. Effects of environmental conditions on *Lm* and *Arcobacter* prevalence

**FIGURE 4:**
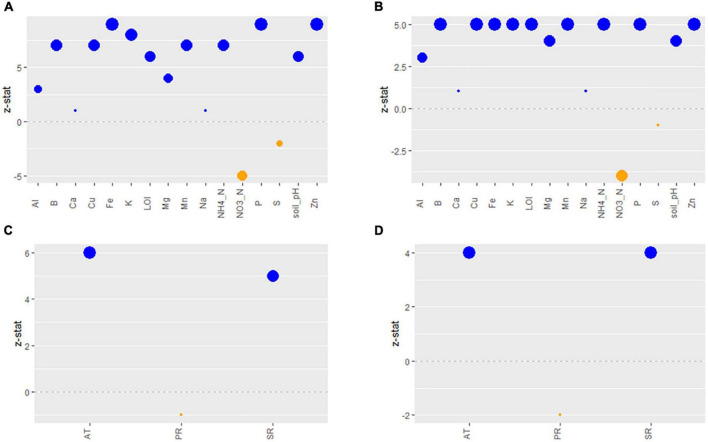
Effect sizes (z-statistic) of the observed soil chemistry variables composing the predicted (standardized) soil fertility latent variable for the *Lm* **(A)** and *Arcobacter* **(B)** models. Effect sizes (z-statistic) of the observed weather variables composing the predicted (standardized) Weather latent variable for the *Lm* **(C)** and *Arcobacter* **(D)** models. Blue dots are positive effect sizes and orange dots are negative effect sizes. The larger the dot, the larger the effect size, i.e., the more important the indicator. Al, aluminum; B, boron; Ca, calcium; Cu, copper; Fe, iron; K, potassium; Mg, magnesium; Mn, manganese; Na, sodium; P, phosphorus; S, sulfur; Zn, zinc; LOI, loss-on-ignition; AT, 24 h average of air temperature; PR, precipitation; SR, solar radiation.

Most soil characteristics analyzed in OH soil samples were good indicators of soil fertility, as defined in the methods, i.e., had large effect sizes (large, standardized regression coefficients), with the exceptions of Ca, Na, and S. Soil characteristics with larger size effects were considered as good indicators of soil fertility. All indicators had positive loadings, i.e., increasing values indicated increasing “fertility,” except for NO_3_-N and S, which had negative loadings, i.e., increasing NO_3_-N and S indicated decreasing fertility (Figure 4).

**FIGURE 5:**
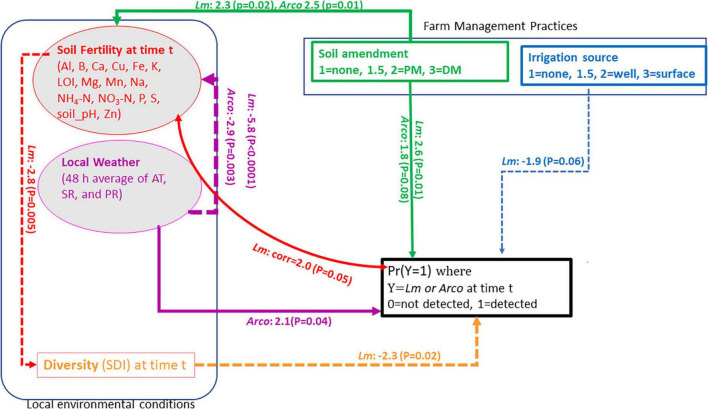
Final (parsimonious) model for OH soil *Lm* and *Arcobacter* (*Arco*) prevalence. Numbers on single-headed arrows are the standardized regression path estimates (*z*-statistic) and *p*-values (in parentheses). The thickness of the arrow reflects the strength of the relationship, with solid-line arrows indicating positive relationships and dash-line arrows indicating negative relationships. For plot simplicity, the soil nutrient covariances are provided in Supplementary Figure 2. Al, aluminum; B, boron; Ca, calcium; Cu, copper; Fe, iron; K, potassium; Mg, magnesium; Mn, manganese; Na, sodium; P, phosphorus; S, sulfur; Zn, zinc; LOI, loss-on-ignition; PM, poultry manure; DM, dairy manure; AT, air temperature; SR, solar radiation; PR, precipitation; SDI, Simpson’s diversity index.

**FIGURE 6:**
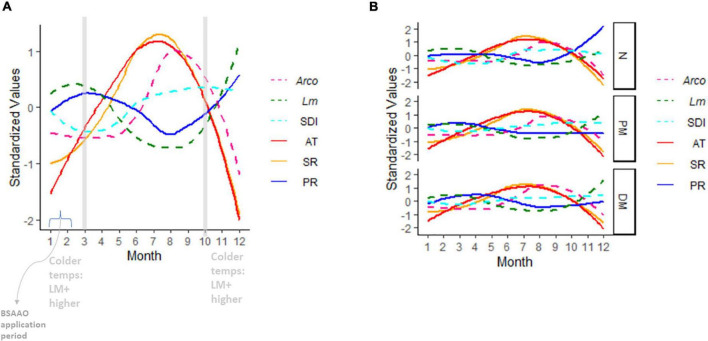
Seasonal trends in OH air temperature (AT), precipitation (PR), and solar radiation (SR). Overlaid are *Lm* soil prevalence, *Arcobacter* (*Arco*) prevalence, and Simpson’s diversity index (SDI) in OH soil. AT, PR, and SR are 48 h moving averages. All variables are standardized to the same scale using the R standardize function in the robustHD package (Alfons, 2019). Soil amendment groups are aggregated in **(A)** and grouped in **(B)**. BSA, biological soil amendment; BSAAO, biological soil amendments of animal origin; PM, poultry manure; DM, dairy manure; N, not amended with BSA or BSAAO; AT, 24 h average of air temperature; SR, solar radiation; PR, precipitation; SDI, Simpson’s diversity index.

The strongest indicators of local weather’s ability to predict *Lm* and *Arcobacter* prevalence in OH soil were AT and SR (Figures 4C, D), which is expected given seasonal patterns associated with changes in temperature due to increased solar radiation. AT and WS were significant indicators in predicting *Lm* prevalence in GA soil but SR and soil amendments were not (Figure 2). *Lm* prevalence in GA soil was directly impacted by weather (*P*_*GA*_ = 0.04; Figure 2B) and, in OH, the impact of weather on soil *Lm* prevalence was mediated by soil fertility, with decreasing fertility correlating with decreasing *Lm* prevalence (*P* = 0.05; Figure 5). Observed *Lm* prevalence was highest during the coolest part of the year, winter and spring (Figure 6A and Supplementary Figure 1). *Arcobacter* prevalence in OH soil was directly impacted by weather (*P* = 0.04; Figure 5) but in an opposite direction, with observed *Arcobacter* prevalence highest during the warmest part of the year (summer and fall) (Figure 6A). The observed effect of PR was not nearly as prominent as AT or SR in OH or GA (effect size*_*Lm*,_* = 0 in GA, effect size*_*Lm*,_* = −1 and effect size*_*Arcobacter*_* = −2 in OH).

OH soil fertility itself was significantly impacted by both soil amendment (*P*_*Lm*_ = 0.02, *P*_*Arcobacter*_ = 0.01) and local weather (*P*_*Lm*_ < 0.0001, *P*_*Arcobacter*_ = 0.003; Figure 5). Soil fertility was highest when a DM-BSAAO was used, followed by PM-BSAAO. Increasing AT and SR and, to a lesser extent, decreasing PR were correlated with decreasing soil fertility in the summer. Soil fertility was highest in winter when AT was low and PR was largely in the form of snow and ice. Altogether, these factors contributed to improved soil fertility in the winter. AT and SR had a more pronounced effect on soil fertility than PR (Figure 4C and D). Soil fertility had a correlative effect on *Lm* but not *Arcobacter* prevalence, but had a direct effect on diversity (*P*_*Lm*_ = 0.005; Figure 5) in OH, where diversity decreased as soil fertility increased. Decreasing diversity, in turn, was correlated with higher *Lm* prevalence (*P* = 0.02), indicating an indirect effect of soil fertility on *Lm* prevalence. These results were in line with the expected results, suggesting the observations that informed the model overall were likely consistent with environmental factors.

**FIGURE 7:**
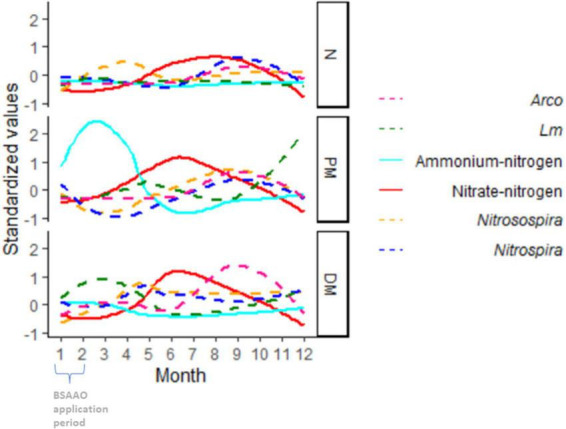
Seasonal Trends in *Lm* and *Arcobacter* (*Arco*) prevalence, *Nitrosospira* relative abundance, *Nitrospira* relative abundance, ammonium-nitrogen and nitrate-nitrogen levels in Ohio soil, grouped by soil amendment. All variables are standardized to the same scale using the R standardize function in the robustHD package. BSA, biological soil amendment; BSAAO, biological soil amendments of animal origin; PM, poultry manure; DM, dairy manure; N, not amended with BSA or BSAAO.

While the role of plant nitrogen utilization could not be properly evaluated in this study, some intriguing patterns were observed between the levels of ammonium and nitrate, local weather, and soil amendment in OH with *Lm* showing an increased prevalence late in the year in PM-amended fields compared to DM- and non-BSAAO-amended fields (Figure 7). Evaluation of the presence of *Nitrospira* and *Nitrosospira*, along with *Lm* prevalence, nitrate levels, and ammonium levels, showed a potential relationship between these factors, possibly initiated by the large increase in ammonium levels observed only in PM-amended fields (Figure 7). Two genera associated with nitrification are *Nitrospira* and *Nitrosospira* (Daims, 2014). Their role in nitrogen processing may be affected by the nitrogen source provided based on the use of DM or PM, the latter with a higher content of ammonium Herbert et al.,^2^ and could explain the observed inverse relationship between the levels of ammonium (high to low) and nitrate (low to high) during the growing season in OH (Figure 7). PM, rich in urea and uric acids, was shown to boost ammonium levels, as expected, when (Figure 7). In these fields, an increase in *Lm* prevalence was observed late in the year, but not in non-BSAAO-amended fields, and was lower in DM-amended fields (Figures 6B, 7). Additionally, there are marked differences between these field groups when evaluating the relative abundance of *Nitrospira* and *Nitrosospira*.

**FIGURE 8:**
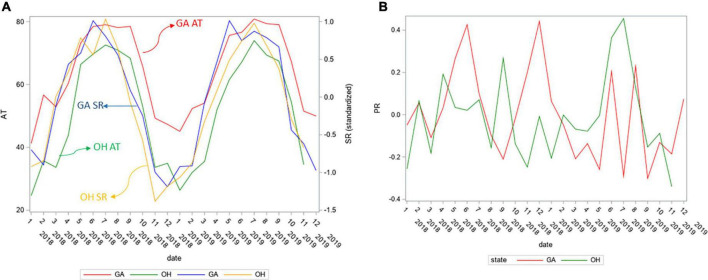
**(A)** GA and OH monthly average air temperature (AT) and average solar radiation (SR) for study years 2018 and 2019. **(B)** GA and OH monthly average precipitation (PR) for study years 2018 and 2019. SR and PR were standardized to the same scale as they were measured differently in GA and OH.

To concentrate on regional differences in *Lm* soil prevalence, possibly due to different weather patterns, the GA and OH weather was compared. The AT and SR values for GA and OH (Figure 8A), and PR data for OH (Figure 8B) were cross-plotted. PR trends were similar cross year (2018, 2019) and were analogous cross both states (GA, OH). The SR trends were also similar cross year (2018, 2019) but the GA SR peaked around May, about two months before the OH SR peaked, around July. To ascertain when the prevalence of *Lm* in soil first significantly decreased, a structural change analysis was conducted. The mean monthly *Lm* prevalence in OH soil was calculated, over both years aggregated. Unlike OH, where soil was sampled throughout the year, the soil in GA was only sampled March through October, due to different amendment application times, resulting in insufficient longitudinal samples to conduct a structural change analysis in GA as mentioned earlier for other analyses.

The Quandt Likelihood Ratio (QLR) statistic (Quandt, 1960) was used to detect the presence of structural breaks in the OH data and was calculated using the *Fstats* function in the strucchange R package (Zeileis et al., 2002, 2003). To determine when the structural break(s) occurred, the *breakpoints* function in the same R package was used. The QLR tests detects structural changes of a regression of the *Lm* soil prevalence at month j regressed on the previous month’s (month j-1) prevalence, i.e., detects month-to-month prevalence changes. Only one structural break in OH was detected in March (*P*_QLR_ = 0.05) and is evident in Figure 6A.

#### 3.3.2. *Listeria monocytogenes* and *Arcobacter*

**FIGURE 9:**
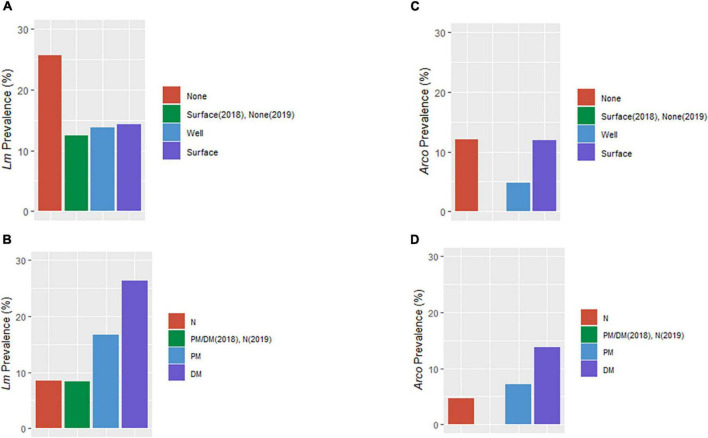
Distribution of *Lm* and *Arcobacter* (*Arco*) prevalence in OH soil by irrigation source **(A,C)** and soil amendment **(B,D)**. BSA, biological soil amendment; BSAAO, biological soil amendments of animal origin; None, no irrigation of the field; Surface, irrigation performed with surface water; Well, irrigation *via* well water; N, not amended with BSA or BSAAO; PM, poultry manure; DM, dairy manure.

The final parsimonious *Lm* and *Arcobacter* model for OH revealed a causality effect associated with the type of soil amendment (*P*_*Lm*_ = 0.01, *P*_*Arcobacter*_ = 0.08) with prevalence highest for DM-amended fields (Figures 5, 9). Although the direct effect of soil amendment on *Lm* prevalence in GA was not significant, the observed *Lm* prevalence in PM-amended fields was marginally higher than for green compost-amended fields. A causal effect of irrigation source in OH (*P* = 0.06) on *Lm* prevalence was observed with prevalence highest for non-irrigated OH fields (Figures 5, 9A). Although, in OH, surface water samples presented the highest observed *Lm* prevalence and well water samples presented zero observed *Lm* prevalence (Table 2), soil samples from surface water-irrigated fields had similar *Lm* prevalence to well-irrigated fields, while the soil samples from non-irrigated fields had the highest *Lm* prevalence (Figure 9A). Since the decision regarding whether to irrigate may be influenced by known on-farm risk factors, such as resident animals, amendment practices were evaluated to determine if they might influence irrigation practices. When evaluating whether amendment choice might correlate with irrigation choice, it was found that: PM-amended fields were equally likely to be irrigated with surface or well water; DM-amended fields were more likely to be irrigated with well water; and non-amended fields were more likely to not be irrigated.

#### 3.3.3. Diversity of bacterial community in soil

Metataxonomic sequence analysis was performed for 305 of the 311 OH soil samples. For purposes of informing the model, samples that were positive for *Lm* after enrichment (culture positive) were considered positive for *Lm*, even if the culture-independent sequencing results did not indicate the presence of *Lm*. In fact, an evaluation of the sensitivity of microbiome metataxonomics when detecting *Lm* in soil samples, only two OH soil samples were found to contain *Lm* by 16S rRNA sequencing analysis whereas 45 OH soil samples were culture-positive for *Lm*. Additionally, the two samples from which *Lm* levels were detected by metataxonomics, were culture-negative for *Lm*. These data highlight the need for culture enrichment of foodborne pathogens from environmental samples. Furthermore, the two samples which were *Lm* culture-negative but contained detectable levels of *Lm* in the metataxonomic analysis were included as *Lm*-containing samples in the analysis. The detection of *Arcobacter* using metataxonomics was also incongruous with culture results. Four OH soil samples had levels of *Arcobacter* detected by metataxonomics, three of which were culture-negative for *Arcobacter* and one of which was not cultured for *Arcobacter*. Of the 21 OH soil samples, which were culture-positive for *Arcobacter*, none had levels of *Arcobacter* detected by 16S rRNA gene amplicon sequencing. These 21 samples were included in the analysis as containing *Arcobacter* and the *Arcobacter* relative abundance (percent hit) was set to half the minimum relative abundance of the four *Arcobacter* containing metataxonomic samples, 0.002.

Diversity of the microbiome was calculated at the family level with the SDI ranging from 0.89 to 0.98. The type of soil amendment did not appear to influence bacterial diversity (Figure 6B). Although weather did not have a direct impact on bacterial diversity, diversity was visibly lower in the winter through spring, reaching its nadir in April/May (Figure 6A). This observation indicated that April/May represented an inflection point in bacterial diversity, perhaps due to increased temperatures that were disadvantageous for cold-adapted bacteria, while growth of more warm-adapted microorganisms had not yet occurred to levels detectable by metataxonomics. Diversity increased significantly with decreasing soil fertility in the *Lm* model (*P*_*Lm*_ = 0.005; Figure 5). As previously mentioned, fertility decreased with increasing AT and SR; as such, the effect of weather on diversity appeared mediated through soil fertility.

#### 3.3.4. Co-occurrence in OH soil

**FIGURE 10:**
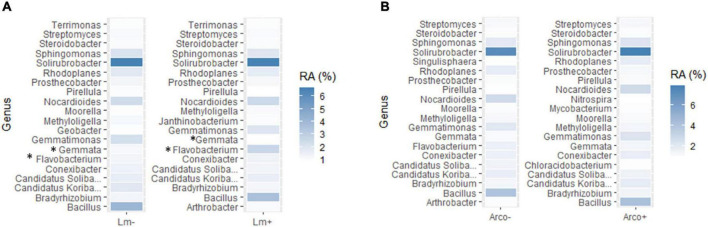
**(A)** Top (most abundant) twenty genera detected (16S) in *Lm* negative (–) samples vs. top 20 genera detected in *Lm* positive (+) samples in OH soil. **(B)** Top twenty genera detected in *Arcobacter* negative (–) samples vs. top 20 genera detected in *Arcobacter* positive (+) samples in OH soil. The darker the color, the more abundant the genus, i.e., the higher the relative abundance (%). An asterisk (*) denotes genera with significantly different mean relative abundance between positive and negative samples. RA, mean relative abundance.

**FIGURE 11:**
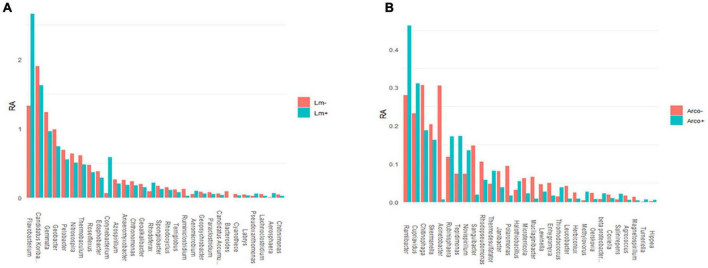
Top thirty genera detected (16S) with significant differences between their mean relative abundance (%) when comparing *Arcobacter* negative (–) and positive (+) OH soil samples **(A)** and between *Lm* negative (–) and *Lm* positive (+) OH soil samples **(B)**. Genera names were truncated at twenty characters. *Bacteriodes* RA in Lm+ samples was 0.000883. RA, mean relative abundance.

The top twenty most abundant genera in *Lm* and *Arcobacter* negative (–) samples were compared to the top twenty most abundant genera detected in *Lm* and *Arcobacter* positive (+) samples (Figure 10). In general, the top twenty genera are similarly abundant in positive and negative samples for *Lm* and *Arcobacter* (Figure 10) with two exceptions, mentioned below. As such, the profiles of dominant genera in soil appeared similar and their influence on pathogen prevalence did not appear substantial. The relative abundances of *Flavobacterium* and *Gemmata*, however, were significantly different (i.e., non-overlapping 95% CI, Table 3) between *Lm+* and *Lm*-samples, with the former more abundant in *Lm+* samples and the latter more abundant in *Lm*- samples. Regarding the genera with significantly different mean relative abundances between pathogen negative and positive samples in the *Arcobacter* co-occurrence analysis, the mean relative abundances (Figure 11A) were much lower (max∼0.5) than the mean relative abundances for *Lm* co-occurrence analysis (Figure 11B), max = 2.5); this signifies that for *Arcobacter* the genera differences observed between positive and negative samples were generated by genera that represented a smaller proportion of the soil microbiome than those genera associated with *Lm* presence or absence.

**TABLE 3:** Top thirty genera whose mean relative abundance was most significantly different between (A) samples with no detectable *Lm* (*Lm*-) and samples with detectable *Lm* (*Lm*+) and (B) samples with no detectable *Arcobacter* (*Arco*-) and samples with detectable *Arcobacter* (*Arco*+).

A							B						
	*Lm*+			*Lm*-				*Arco*+			*Arco*-		
Genus	Mean	Lower CL	Upper CL	Mean	Lower CL	Upper CL	Genus	Mean	Lower CL	Upper CL	Mean	Lower CL	Upper CL
*Aeromicrobium*	0.0945	0.0745	0.1589	0.0524	0.0451	0.0595	*Acinetobacter*	0.0063	0.0030	0.0129	0.3052	0.1552	1.3559
*Aerosphaera*	0.0669	0.0238	0.4378	0.0065	0.0027	0.0194	*Agrococcus*	0.0057	0.0019	0.0131	0.0169	0.0137	0.0227
*Anaeromyxobacter*	0.1799	0.1457	0.2210	0.2572	0.2355	0.2864	*beta proteobacter.*	0.0223	0.0129	0.0480	0.0071	0.0048	0.0106
*Azospirillum*	0.2054	0.1701	0.2372	0.2627	0.2461	0.2831	*Chitinophaga*	0.1874	0.1535	0.2548	0.3065	0.2602	0.4093
*Bacteroides*	0.0009	0.0003	0.0027	0.0889	0.0223	1.0358	*Coxiella*	0.0098	0.0060	0.0148	0.0191	0.0155	0.0222
*Candidatus Accumu.*	0.0382	0.0323	0.0459	0.0559	0.0475	0.0681	*Cupriavidus*	0.3111	0.2750	0.3656	0.2323	0.2189	0.2463
*Candidatus Koriba.*	1.6213	1.4506	1.8140	1.8997	1.8285	1.9825	*Enhygromyxa*	0.0171	0.0081	0.0263	0.0503	0.0338	0.0958
*Chitinimonas*	0.0272	0.0209	0.0352	0.0416	0.0378	0.0469	*Halothiobacillus*	0.0540	0.0382	0.0694	0.0319	0.0281	0.0360
*Chthonomonas*	0.1749	0.1479	0.2040	0.2384	0.2278	0.2526	*Herbiconiux*	0.0088	0.0050	0.0180	0.0249	0.0195	0.0323
*Corynebacterium*	0.5816	0.1749	2.2222	0.0662	0.0455	0.1005	*Hippea*	0.0054	0.0034	0.0090	0.0017	0.0013	0.0024
*Cyanothece*	0.0291	0.0186	0.0414	0.0544	0.0449	0.0730	*Janibacter*	0.0378	0.0288	0.0471	0.0808	0.0644	0.1051
*Edaphobacter*	0.2888	0.2295	0.3304	0.3829	0.3605	0.4106	*Leucobacter*	0.0085	0.0043	0.0147	0.0417	0.0296	0.0825
*Flavobacterium*	2.6550	1.7939	3.9411	1.3286	1.0864	1.6932	*Lewinella*	0.0268	0.0140	0.0392	0.0464	0.0392	0.0632
*Gemmata*	0.9593	0.8502	1.1113	1.2336	1.1648	1.3119	*Magnetospirillum*	0.0044	0.0024	0.0074	0.0133	0.0102	0.0157
*Geoalkalibacter*	0.1516	0.1210	0.1856	0.1960	0.1872	0.2091	*Methylovorus*	0.0274	0.0070	0.4757	0.0039	0.0032	0.0057
*Geobacter*	0.7395	0.5791	0.9095	0.9872	0.9373	1.0455	*Microterricola*	0.0228	0.0145	0.0346	0.0625	0.0470	0.0804
*Geopsychrobacter*	0.0584	0.0491	0.0676	0.0820	0.0763	0.0885	*Mucilaginibacter*	0.0090	0.0035	0.0250	0.0660	0.0500	0.1124
*Labrys*	0.0341	0.0279	0.0420	0.0474	0.0432	0.0576	*Novispirillum*	0.1357	0.0937	0.1881	0.0739	0.0658	0.0813
*Lachnoclostridium*	0.0232	0.0174	0.0311	0.0518	0.0324	0.0926	*Oerskovia*	0.0074	0.0043	0.0134	0.0231	0.0146	0.0379
*Nitrosospira*	0.5063	0.4379	0.5947	0.6386	0.6055	0.6780	*Polaromonas*	0.0171	0.0054	0.0460	0.0944	0.0784	0.1305
*Paraclostridium*	0.0499	0.0395	0.0605	0.0782	0.0638	0.0989	*Ramlibacter*	0.4620	0.3519	0.5496	0.2794	0.2470	0.3084
*Pelobacter*	0.5541	0.4913	0.6278	0.6886	0.6513	0.7335	*Rhodopseudomonas*	0.0578	0.0401	0.0684	0.1059	0.0970	0.1155
*Pseudoxanthomonas*	0.0550	0.0351	0.0882	0.0255	0.0198	0.0301	*Rubinisphaera*	0.1719	0.1428	0.2007	0.1181	0.1083	0.1269
*Rhodocyclus*	0.1117	0.0912	0.1407	0.1495	0.1409	0.1574	*Salinirepens*	0.0209	0.0141	0.0330	0.0069	0.0056	0.0084
*Rhodoferax*	0.2179	0.1186	0.4360	0.0879	0.0804	0.1029	*Sanguibacter*	0.0194	0.0091	0.0586	0.1479	0.0671	0.4369
*Roseiflexus*	0.3678	0.3107	0.4119	0.4701	0.4288	0.5204	*Skermanella*	0.1631	0.1376	0.1893	0.2035	0.1893	0.2168
*Ruminiclostridium*	0.0268	0.0191	0.0355	0.1216	0.0523	0.6547	*Tepidimonas*	0.1730	0.1096	0.2612	0.0735	0.0617	0.0945
*Spongiibacter*	0.1220	0.1017	0.1511	0.1668	0.1519	0.1858	*Thermodesulfatator*	0.0819	0.0602	0.1164	0.0477	0.0418	0.0532
*Terriglobus*	0.0754	0.0562	0.0922	0.1169	0.1091	0.1284	*Thiorhodococcus*	0.0388	0.0222	0.0661	0.0147	0.0111	0.0182
*Thermobaculum*	0.4798	0.4055	0.5699	0.6090	0.5878	0.6370	*Turneriella*	0.0067	0.0035	0.0134	0.0012	0.0008	0.0017

Lower CL, lower limit of 95% CI; Upper CL, upper limit of 95% CI; CL, confidence limit.

## 4. Discussion

### 4.1. Pathogen prevalence in dairy manure

While green compost, PM, and DM were evaluated in this study, there was a higher prevalence of the evaluated pathogens in DM. Additionally, as green compost and PM were only collected around the time of application, analysis of seasonality trends was not possible. However, due to the collection of DM throughout the growing season, it was possible to determine the prevalence data for *Arcobacter* and *Lm* and evaluate potential patterns associated with season, leading to a possible explanation for the observed pattern. In the spring and summer, dairy herds associated with this study shifted to grazing in pastures around the farm. A similar shift likely occurred at other farms in the region. As a result, there was likely increased interaction of cattle with surface water that could lead to transmission of *Lm* to or from neighboring animals. This exposure could be by drinking contaminated surface water, which could occur independent of pasture access, as well as by the animals entering surface water that cross through the pasture, transmitting pathogens to and from pastures. This increased potential for exposure and transmission could explain the observed increase in *Lm* prevalence in OH DM in the spring that continued through the fall but declined in the winter (Figure 3) when herds were generally kept within barns, which could potentially reduce direct interaction with surface water. However, the possibility remains that dietary shifts associated with these seasonal shifts could be affecting *Lm* prevalence in DM as well. Although *Lm* DM prevalence was lowest in the winter, the regional herd, encompassing all cattle evaluated for *Lm via* manure testing, carried some level of *Lm* year-round. This presence of *Lm* in some cattle within the region would provide an opportunity for re-introduction of *Lm* to the agricultural region in the spring. Higher *Lm* prevalence in DM and surface water (Figure 3) samples during the summer was not observed in OH soil (Figure 6A). Soil *Lm* prevalence, instead, was higher in the spring than the summer. This reduced prevalence could be due to reduced runoff in the summer as precipitation was lowest in the summer and/or increased competition with other soil organisms in the warmer months. Similarly, *Arcobacter* prevalence in DM was much higher in warmer months than in the winter. Others have similarly reported higher frequency of *Arcobacter* in feces from cows in southern dairy herds than in those in the northern states, which may reflect higher transmission in warmer climates (Wesley et al., 2000).

### 4.2. Pathogen prevalence in water

Surface water had a higher pathogen prevalence when compared to well water. Although some ponds and streams evaluated were not used directly for agricultural irrigation, their relatively high *Lm* and *Arcobacter* prevalence should be considered since they could still be sources of pathogen introduction. Pathogens could be introduced into the field *via* animal movement and/or precipitation events could lead to flooding of surface water, introducing contaminated water into the field. This study did not examine surface water during the winter since it was not used for irrigation. However, an assessment of *Lm* prevalence in winter water samples could provide better information about how *Lm* survives and circulates in an agricultural region and how those factors contribute to the potential for *Lm* contamination of fresh produce. This is of particular interest after the observations of *Lm* prevalence in DM, indicating the potential for circulation between the regional herd and surface water. *Lm* prevalence in OH surface water was observed to peak in the summer when the domestic animals were more likely to be outside with possible direct interaction with surface water. This prevalence decreased to undetectable levels in the fall (Figure 3). Unlike *Lm* prevalence, *Arcobacter* presence in surface water was not significantly different in spring, summer and fall seasons. Similarly, others have shown that the prevalence of *Arcobacter* and five associated virulence genes in water samples from various sources were not impacted by weather in the Kathmandu Valley in Nepal (Ghaju Shrestha et al., 2019). These results, revealing differences in how each pathogen survives and persists in the agricultural environment, highlighted the need to consider pathogen-specific risk assessment models and mitigation strategies in agricultural practices.

### 4.3. Pathogen prevalence in soil

The SEM approach used to evaluate factors associated with pathogen prevalence in soil is in line with the exploratory and confirmatory attitude of this study. Lam and Maguire provided a good description of SEM and its uses (Lam and Maguire, 2012). *Lm* and *Arcobacter* were both directly impacted by soil amendment, with pathogen prevalence being highest for DM amendment, second highest for PM amendment and lowest for no amendment (Figures 5, 9B, D). *Lm* presence in non-amended fields was marginally higher than *Arcobacter* presence (Table 2), suggesting *Arcobacter* prevalence in soil was primarily driven by BSAAO usage. BSAAO usage may serve as either the vehicle for *Arcobacter* introduction or provide a more favorable environment for pathogen survival. Other factors (e.g., irrigation source) appeared to have critical influence on *Lm* prevalence, though BSAAO usage did contribute to *Lm* prevalence. BSAAO serving as a primary driver for *Arcobacter* prevalence may explain the differences in prevalence observed between GA and OH farms in this study, with the former having reduced BSAAO usage and no use of DM and a concurrent reduction in *Arcobacter* prevalence. It is intriguing that this difference was also seen in surface water, suggesting added factors may be reducing *Arcobacter* prevalence in GA though it could simply be the absence of introduction from reduced BSAAO applications, potentially limited to the lack of DM.

*Lm* prevalence in soil was highest during the winter and spring when AT was lowest and precipitation amounts were marginally higher (Figures 6A, B). The impact of increasing AT (|effect size| ∼6) on decreasing *Lm* prevalence appeared more salient than the impact of increasing PR (|effect size| ∼1) on increasing *Lm* prevalence (Figure 4C). Although this study found, as others did (Strawn et al., 2013a,b; Weller et al., 2015a; Falardeau et al., 2018; Harrand et al., 2020), that *Lm* positivity increased with increased rainfall or soil moisture, AT appeared to suppress *Lm* more (i.e., higher effect size) than the moisture supported *Lm*. This observation is intriguing however, as GA weather had higher AT and similar PR compared to OH, but GA *Lm* prevalence was only slightly lower than that of OH in both soil and water samples. This suggests the possibility that AT alone may not be suppressing *Lm* or that temperatures during winter may provide a basal level of support for *Lm* prevalence throughout the year. However, it may also be that the microbiome population dynamics or other factors, such as soil texture, may vary between the different regions resulting in equivalent *Lm* prevalence despite the differences in temperature. SDI was highest during the summer and fall (Figure 6A), the same seasons where *Lm* prevalence is at its lowest, and the model (Figure 5) showed a direct role for diversity in the reduction of *Lm*, suggesting that competition played a role in suppression of *Lm*. It should be considered that AT, therefore, could impact *Lm* directly and indirectly.

Weather had a stronger direct effect on *Arcobacter* with prevalence increasing as AT increased and PR decreased (Figure 6A). A predictive model to measure the growth rate of *A. butzleri* showed that growth was directly proportional to increasing temperature, achieving maximum growth at a storage temperature of 40^°^C (Park and Ha, 2015). A more recent study has shown optimal growth of the three major human pathogenic *Arcobacter* species (*A. butzleri*, *A. cryaerophilus*, and *A. skirrowii*) at 35°C, followed by growth on agar plates at 30°C under microaerophilic conditions (Nguyen et al., 2021). Given average temperatures never exceeded 95°F (35°C), these prior data support the findings of this study, which indicated warmer temperatures supported *Arcobacter* prevalence, potentially by improved growth.

Soil fertility was directly impacted by both weather and soil amendment, as expected. Soil fertility decreased with rising AT and decreasing PR and was highest in DM-amended fields, with a slight decrease in PM-amended fields and a substantial decrease in non-amended fields. While some elements (anions) in the fertility panel may naturally leach out of soil through water percolating through soil column or surface erosion (water/wind whose speed increased through the growing season), all the elements in the panel are affected by weather through plant/crop uptake. Greater AT and SR enhance crop growth and thereby nutrient consumption, resulting in decreased soil fertility as the growing season progresses. While soil fertility was only correlated with *Lm* prevalence, the improvement of soil fertility associated with soil amendment usage could have indirectly affected *Lm* and *Arcobacter* prevalence *via* improved nutrient content. Although there are no studies to test the effect of soil fertility on the prevalence of *Arcobacter*, it is important to recognize its global presence in water bodies and various animals, including food and farm animals, domestic birds, wildlife, and zoo animals (Hsu and Lee, 2015).

The preliminary assessment, evaluating NO_3_-N and NH_4_-N separate from soil fertility, indicated the possibility of a relationship between ammonium, nitrate, *Nitrospira*, *Nitrosospira*, and *Lm* prevalence, though this observation requires further analysis since AT or other variables could play a role as well. However, within this study, given that manure was applied at a set time in the year and AT varies throughout the year and correlates with the season, it was not possible to disconnect these variables to evaluate their impact on nitrifying bacteria and *Lm* prevalence independently. The observed increase in *Lm* prevalence in PM-amended fields late in the year suggested the possibility that the ammonium increase associated with PM might impact nitrifying microorganisms, with one nitrifying genus, *Nitrosospira*, found to have a significant difference in relative abundance between *Lm*+ and *Lm*- fields (Figure 11A).

There are hypotheses that a healthy, robust soil, i.e., high soil fertility, may limit pathogen prevalence (Devarajan et al., 2021; Jayaraman et al., 2021). In fact, one recent study found that the use of cover crops and compost boosted soil diversity, improving suppression of *Salmonella* and *Lm* (Devarajan et al., 2021). However, in our study, while we found soils with higher SDI had reduced *Lm* prevalence, soils with higher soil fertility, which was improved with the use of BSAAO, which included both raw and composted manure, had a decreased SDI (Figure 5 and 6A). Additionally, the use of BSAAO was associated with an increased prevalence of *Arcobacter* and *Lm*, suggesting added complexity within these relationships. In particular, the *Lm* model showed that increased soil fertility resulted in decreased SDI, which could indirectly increase *Lm* prevalence as decreased SDI was correlated with higher *Lm* prevalence, though the same was not true for *Arcobacter* (Figure 5). Therefore, careful consideration of the attributes of a pathogen is important when testing how farm management practices could impact its prevalence.

This finding that there could be differing impacts on pathogens was further highlighted when evaluating the impact of irrigation on pathogen prevalence. Irrigation was found to have no impact on *Arcobacter* prevalence (Figures 5, 9C). However, irrigation source had a direct impact on *Lm* prevalence, though the result was counter to the expected results. *Lm* prevalence was highest for non-irrigated fields when compared to both well and surface water-irrigated fields (Figures 5, 9A). One explanation could be that farm management practices could have been impacted by observable risks, such as known proximity to animals, when deciding whether to use surface water for irrigation. Evaluation of farm practices found that non-BSA amended fields were more likely to not be irrigated, suggesting that other factors, such as economics or neighboring animals, may have influenced these decisions. However, metadata to evaluate what factors farmers used to inform their irrigation choice were not available. It is important to note, though, that the soil evaluated was not the soil directly irrigated but adjacent so it is possible that a different observation would have been made closer to the dripline. These possibilities suggest that more studies are needed to understand what could be creating the observed result, given that *Lm* prevalence was highest in surface water samples.

### 4.4. Evaluation of study

SEMs are uniquely suited for (1) identifying relationships (pathways) between latent variables (weather and soil fertility), (2) modeling complex (intermediary) relationships between factors and (3) explicitly identifying covariances between indicators and factors (Nachtigall et al., 2003). SEMs directly estimate both the effect sizes and direction of the effects, simultaneously for all factors, a notable strength of this analysis.

Some factors that indicated possible associations with soil pathogen prevalence but were not included in the soil analyses due to insufficient or incomplete data include comparing organic and non-organic farming, equipment hygiene, insecticide/fungicide application, presence of animal activity, water additives, and soil moisture measurements. The impact of these candidate factors would need a much larger study to be evaluated. Broader and more complete metadata collection was desirable for the study but attaining such on a voluntary basis was challenging, especially with potential for differences in memory to recall specific practices and/or events.

Year to year variability also cannot be eliminated as a factor in this study given 2018 and 2019 were not evaluated to determine if weather related factors were representative of overall weather trends.

## 5. Conclusion

Pathogen prevalence in soil, for both *Lm* and *Arcobacter*, was found to be associated with the use of BSAAO but not the use of surface water as an irrigation source. In fact, the *Lm* model indicated that not irrigating increased *Lm* prevalence, suggesting that other factors that might be linked to the decision to not irrigate, such as animals residing at neighboring farms, wildlife presence, equipment sanitation activities, land topography, or unidentified/unknown variables may be contributing to increased *Lm* prevalence in non-irrigated fields. As noted earlier, BSAAO usage was not correlated with the practice of not irrigating fields. This indirectly suggests that the presence of cattle on farms, which served as the source of DM, did not impact irrigation choices. AT appeared to play a more significant role on pathogen prevalence in soil rather than the use of BSAAO, although the effect of AT was in opposing directions for *Arcobacter* and *Lm*. Additionally, data showed key differences in how environmental conditions impacted pathogen prevalence, indicating that a one size fits all approach to risk mitigation would not control certain pathogens. While the sample size was small and metadata were limited, this real-world comparison of multiple farms in two regions based on BSAAO and irrigation provided critical data to inform and validate controlled studies. The results of this study have identified other factors, i.e., not irrigating could increase pathogen prevalence, that may need further study and the need to better understand how pathogens are established and circulated within the agricultural environment.

## Data availability statement

The data presented in the study are deposited in the NCBI repository, accession number PRJNA894200.

## Author contributions

EL, GR, MK, CG, KJ, LB, CH, KB, LH, UB, BL, and AZ contributed to the conception and design of the study. CH and MF organized the database. JP, MF, CG, and KJ worked on bioinformatics and sequence analysis. MF performed the statistical analysis. CH, LB, CG, UB, LH, BL, AH, KB, JF, MK, KJ, and RR were involved in sample processing. MF, CH, CG, UB, LB, and BL wrote the manuscript. KB was responsible for the revision and proofreading of this work. All authors contributed to the article and approved the submitted version.

## Footnotes

1
https://umaine.edu/soiltestinglab/

2 Herbert, S., Hashemi, M., Chickering-Sears, C., Weis, S. M., Carlevale, J., Campbell-Nelson, K., et al. Conserving Ammonia in Manure. UMass Extension Crops, Dairy, Livestock and Equine Program. Available online at: https://ag.umass.edu/crops-dairylivestock-equine/fact-sheets/conserving-ammonia-in-manure

## Supplementary material

Supplementary Figure 1 Seasonal trends in GA air temperature (AT), precipitation (PR), solar radiation (SR), and wind speed (WS). Overlaid is *Lm* soil prevalence GA soil. AT, PR, SR and WS are 48 h moving averages. All variables are standardized to the same scale using the R *standardize* function in the robustHD package. Soil amendment groups are aggregated in **(A)** and grouped in **(B)**. GC, green compost; PM, poultry manure; AT, 24 h average of air temperature; PR, precipitation; SR, solar radiation; WS, wind speed.

Supplementary Figure 2 Covariances between the soil nutrient covariances included in the OH *Lm* and *Arcobacter* (*Arco*) soil prevalence models. Ca, calcium; Mg, magnesium; Mn, manganese; P, phosphorus; S, sulfur; Zn, zinc; LOI, loss-on-ignition; NO_3__N, nitrate nitrogen; NH_4__N, ammonium nitrogen.
