# Supplementary figures and images for: A longitudinal study to examine the influence of farming practices and environmental factors on pathogen prevalence using structural equation modeling
Source: Front Microbiol. 2023 Apr 6;14:1141043. doi: 10.3389/fmicb.2023.1141043 (PMC10117993; doi:10.3389/fmicb.2023.1141043)

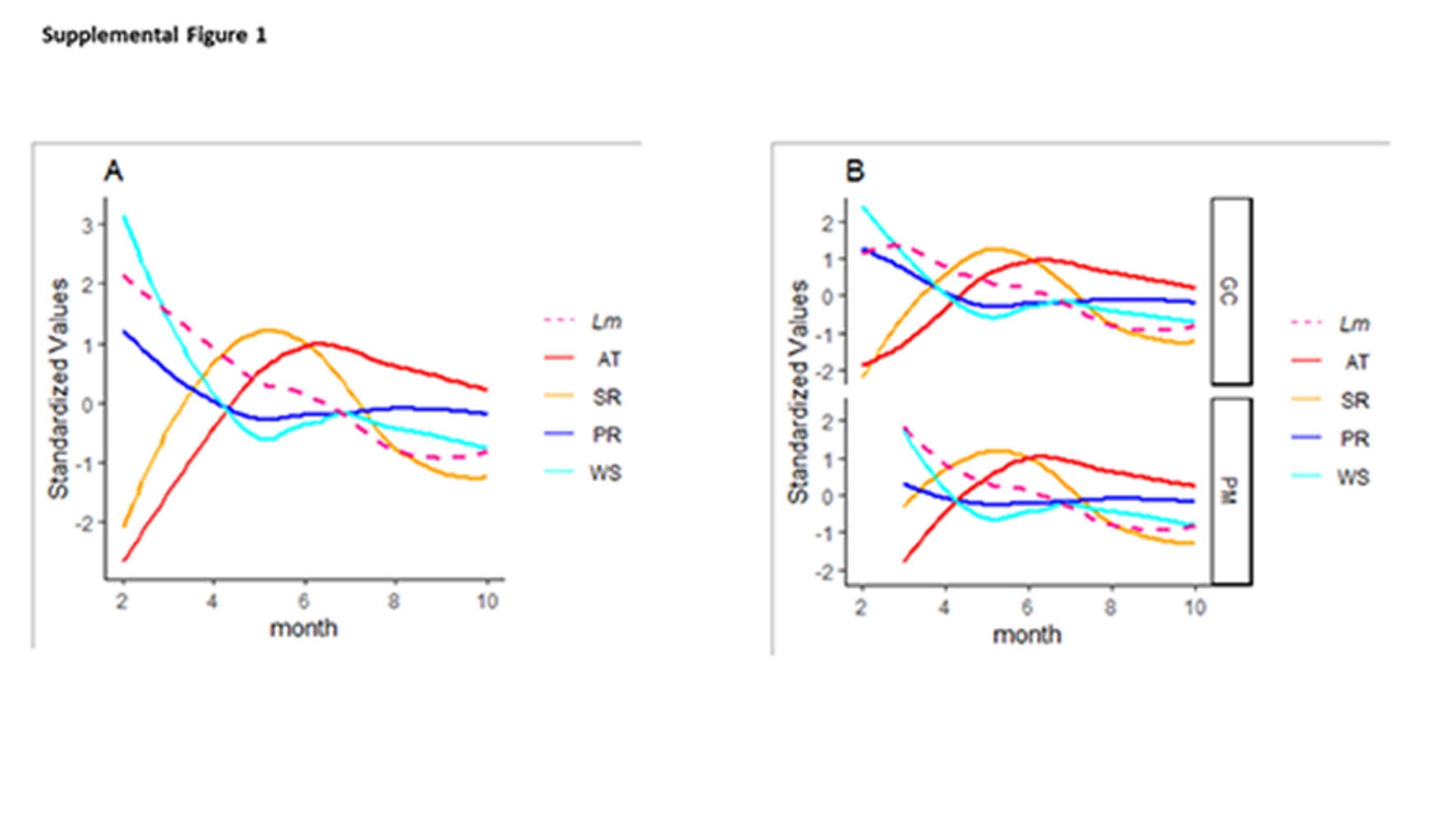

Supplement: Supplementary Figure 1 — Seasonal trends in GA air temperature (AT), precipitation (PR), solar radiation (SR), and wind speed (WS). Overlaid is Lm soil prevalence GA soil. AT, PR, SR and WS are 48 h moving averages. All variables are standardized to the same scale using the R standardize function in the robustHD package. Soil amendment groups are aggregated in (A) and grouped in (B). GC, green compost; PM, poultry manure; AT, 24 h average of air temperature; PR, precipitation; SR, solar radiation; WS, wind speed. [file Image_1.TIF]

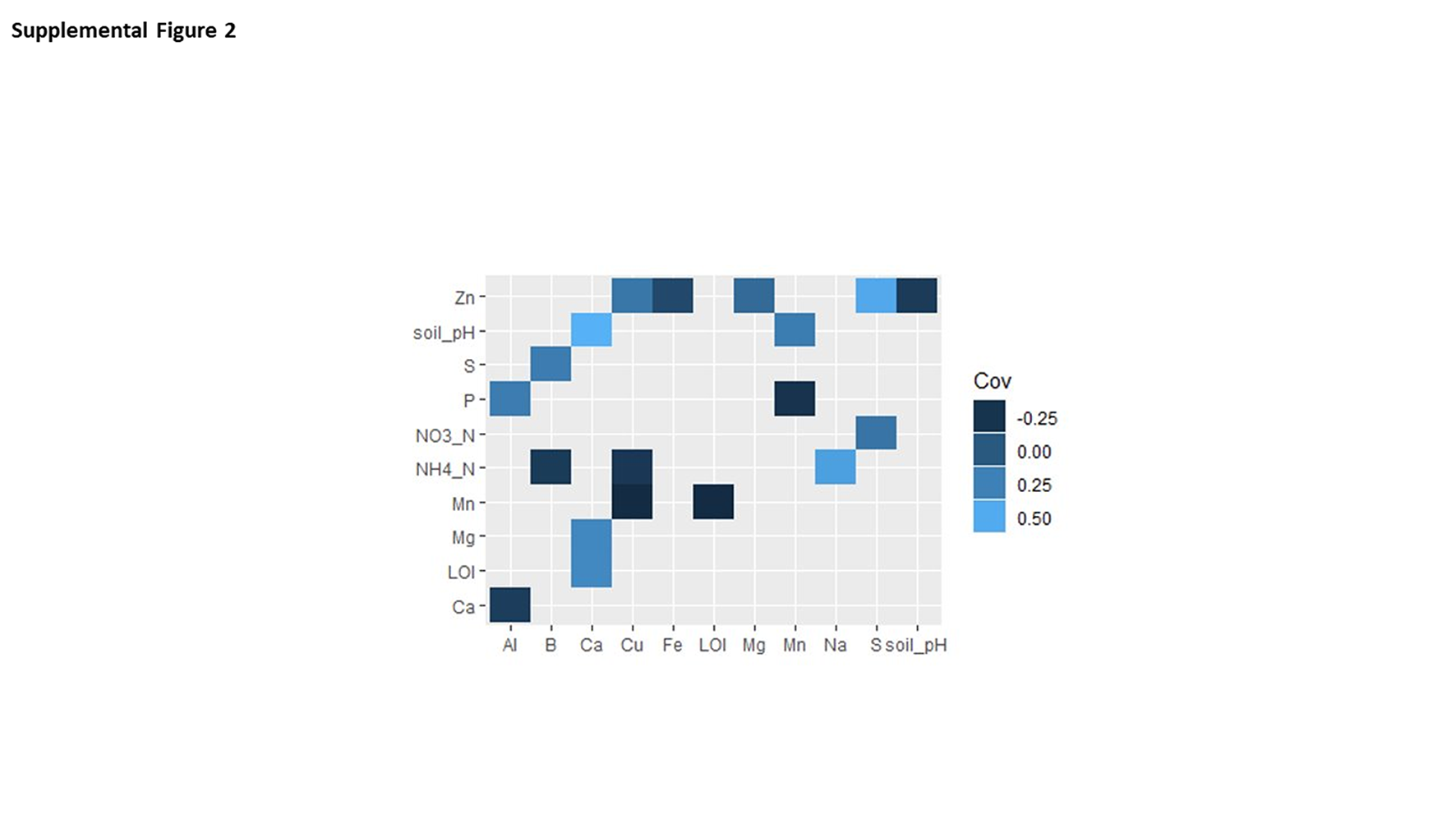

Supplement: Supplementary Figure 2 — Covariances between the soil nutrient covariances included in the OH Lm and Arcobacter (Arco) soil prevalence models. Ca, calcium; Mg, magnesium; Mn, manganese; P, phosphorus; S, sulfur; Zn, zinc; LOI, loss-on-ignition; NO3_N, nitrate nitrogen; NH4_N, ammonium nitrogen. [file Image_2.TIF]
